# Instant Gratification and Overtreating to Be Safe: Perceptions of U.S. Intensive Care Unit Pharmacists and Residents on Antimicrobial Stewardship

**DOI:** 10.3390/antibiotics11091224

**Published:** 2022-09-09

**Authors:** Katharina Rynkiewich, Kruthika Uttla, Leila Hojat

**Affiliations:** 1 Department of Anthropology, Florida Atlantic University, Boca Raton, FL 33431, USA; 2 Department of Anthropology, Case Western Reserve University, Cleveland, OH 44106, USA; 3 Department of Medicine, Division of Infectious Diseases, Case Western Reserve University, Cleveland, OH 44106, USA; 4 Division of Infectious Diseases and HIV Medicine, University Hospitals Cleveland Medical Center, Cleveland, OH 44106, USA

**Keywords:** antimicrobial stewardship, antimicrobial prescribing, practitioner behavior

## Abstract

Antimicrobial stewardship programs have been associated with numerous impacts on medical practice including reductions in costs, antimicrobial resistance, and adverse events. While antimicrobial stewardship is now considered an essential element of medical practice, the understandings of the value of antimicrobial stewardship among medical practitioners vary. Additionally, non-physician practitioners are regularly left out of antimicrobial stewardship interventions targeting antimicrobial decision-making. Here, we contribute the perspective from resident physicians and specialists in pharmacy regarding their involvement in antimicrobial prescribing. Notably, our semi-structured interviews with 10 residents and pharmacy specialists described their limited autonomy in the clinical setting. However, the participants regularly worked alongside primary antimicrobial decision-makers and described feeling pressure to overtreat to be safe. The clear rationales and motivations associated with antimicrobial prescribing have a noticeable impact on physicians in training and non-physician practitioners, and as such, we argue that antimicrobial stewardship interventions targeting primary antimicrobial decision-makers are missing an opportunity to address the breadth of antimicrobial prescribing culture. By looking at the perspectives and rationales of physicians in training and non-physician practitioners, we can see evidence that the act of antimicrobial prescribing is impacted by individuals on all levels of the hierarchies present in medical practice.

## 1. Introduction

The World Health Organization (WHO) and Centers for Disease Control and Prevention (CDC) have identified antimicrobial resistance (AMR) as a multifaceted challenge existing across multiple domains [1,2]. Acknowledging the importance of AMR strategies to combat growing resistance shows that antimicrobial stewardship programs have the potential to reduce costs and improve patient outcomes as well as reduce antimicrobial resistance [3]. Antimicrobial stewardship is now a required element for hospitals in the United States which accept funding from the Centers for Medicare and Medicaid Services (CMS) and is gaining recognition globally as a necessary means of intervening in antimicrobial use patterns.

Following the formal recognition of antimicrobial stewardship programs in the United States, interest and investment in antimicrobial use and stewardship have risen dramatically [4]. However, the ideal antimicrobial stewardship program structure has not been established. Researchers have shown that antimicrobial stewardship interventions tend to focus on changing the behavior of physicians [5,6], yet providing opportunities for pharmacists [7] and nurses [8] to be involved in antimicrobial stewardship is increasingly supported in the literature. Resident physicians and pharmacists, both of whom perform vital functions for the everyday care of hospitalized patients, can sometimes be left out of the fold on antimicrobial stewardship matters. Though they traditionally do not act as the primary decision-makers, residents and pharmacists are well positioned to assist with optimization of antimicrobials prescribed and administered in hospital settings.

Our main objective in this study was to assess both daily practices related to antimicrobial prescribing and levels of awareness regarding the mandate of antimicrobial stewardship to utilize antimicrobials with care within pharmacist and resident groups at institutions where neither is a primary decision-maker for antimicrobial use. Our intention was to use this assessment to advance the understanding of the current perceived role of these groups in antimicrobial stewardship in the United States and to demonstrate how they may be employed more effectively in this capacity. We used a qualitative approach involving semi-structured interviewing to assess how practitioners with limited decision-making power saw their engagement on the themes of antimicrobial prescribing and antimicrobial stewardship. We furthermore chose to focus on the perspectives of residents and pharmacists working in intensive care units (ICUs) to highlight spaces of the hospital where complex patients are regularly cared for by multiple teams necessitating collaboration [9]. In context of our findings, we discuss the potential reasons underlying a potential lack of explicit involvement of residents and specialized pharmacy practitioners in antimicrobial stewardship.

## 2. Materials and Methods

### 2.1. Study Location and Participants

Study participants included pharmacists and residents working in a 1032-bed, academic medical center located in Cleveland, Ohio. Given the size and range of expertise at this location, this facility allowed the study to center around similarly ranked practitioners in multiple ICU spaces within the same institution. The pharmacists included in the study were pharmacy specialists from the medical ICU, trauma-surgical ICU, cardiothoracic ICU, cardiac ICU, and neuro ICU. The residents included anesthesia and critical care residents rotating within the trauma-surgical ICU and cardiothoracic ICU during the study period. The targeted pharmacy and residency groups were not trained specialists in infectious diseases. There are 84 staff and specialty pharmacists working at the site, including 5 specialty pharmacists based in ICUs at the time of the study. There are 55 physicians in the residency program for anesthesia and critical care working at the site, including 24 residents who are typically rotating in ICUs. This site is unique within its geographic region and the following results and conclusions are most fitting for this site.

### 2.2. Methodology

The study was designed by an interdisciplinary research team comprising an infectious diseases specialist (L.H.), a medical anthropologist (K.R.), and a research assistant with training in anthropology (K.U.). A qualitative approach based on ethnographic methods [10] was selected with the intention of the study to uncover perspectives and understandings of physicians in training and practicing pharmacists. In the United States, though hospitals are required by CMS to implement an antimicrobial stewardship program, no specific conditions for these programs are stipulated at the time of this writing. At this study location, following CDC guidance, the antimicrobial stewardship program performs a wide range of services, though most physicians in training may not often have direct exposure to the antimicrobial stewardship team. On the other hand, pharmacists typically interact with antimicrobial stewardship team members regularly. Thus, the study team approached the creation of the semi-structured interview guide by blending questions on the existing antimicrobial stewardship approach at the national level with local contextual factors as identified by study team members based on experience and a short period of observation and discussion. The development of the semi-structured interview guide spanned multiple weeks and was designed to be administered during in-person, one-on-one meetings with a member of the study personnel. The semi-structured interview was designed to be administered in English and take approximately 45–60 min to complete.

### 2.3. Sampling Procedure

A participant list was identified based on role and availability [10]. Work in ICU settings was essential for inclusion in this study. All anesthesia and critical-care residents rotating through the trauma-surgical or cardiothoracic ICUs during June–August 2021 were eligible (N = 24), as were all specialty pharmacists assigned to any ICU during the study period (N = 5). Residents who were not scheduled to work in the ICUs or who were starting their positions during the study period were ineligible. Directors of the ICUs sent an introductory email to all trainees, alerting them to the possibility that they would be invited to join the study. We then recruited participants using email to introduce the study personnel and detail the requested time involved with participation. Follow-up emails were sent one week after the initial invitation. Pharmacists were invited by email directly by the study team. In consideration of the ongoing COVID-19 pandemic, and in agreement with directors of the ICUs, a strategic decision of intentionally limiting follow-up emails was made. Additionally, a cutoff for the study team was established stating that recruitment would end after data saturation to avoid informational redundancy [11]. Thus, after the study team reached five participants in both the resident and pharmacist categories, recruitment was stopped. As we considered the data to be well-saturated with multiple interrelated themes and rich participant description [12] from lengthy interviews, we found no further need to interfere with ICU practice. This method is consistent with conduct of ethnography in medical settings [13].

### 2.4. Ethical Approvals

Ethical approval was obtained from the Institutional Review Board of the participating site (STUDY20210124). Letters of support were obtained from the ICU and pharmacy directors for their approval to include their residents and specialty pharmacists prior to the start of the study. Permission to conduct the study was granted prior to any recruitment efforts on behalf of the study team.

Prior to the start of each interview, an information sheet was read to the participant containing a detailed description of the study approval, team members, and aims. Participants were informed of any benefits and risks associated with the study. The participants were further informed about their right to stop the interview and withdraw from the study at any point. The study team explained the necessity of maintaining confidentiality, anonymity, and voluntarism throughout the research. Verbal informed consent was sought and obtained on the voice recording of the interview.

### 2.5. Analysis

The voice recording of each interview was transcribed by study personnel (K.U.). Transcripts of recorded interviews were read separately by two members of the study team (K.U. and K.R.). Separate lists of potential themes were developed based on an initial read-through. K.U. and K.R. compared lists and solidified initial themes to begin formal qualitative coding. As the initial themes guided qualitative coding analysis [14] using the software NVivo (QSR International (Burlington, MA, USA), accessed in 2021), themes were adjusted and expanded as needed. The focus of this analysis period was to identify key words and frequently used adjectives related to the study topics of antimicrobial stewardship, antimicrobial prescribing, and social dynamics in the ICU. Word frequencies and guided searches based on the initial list of themes were conducted and used to narrow down the focus of the participant responses. The creation of codes gave preference to locally significant terms and descriptors as the study team intended to prioritize an emic approach to understanding local culture.

## 3. Results

Ten in-depth semi-structured interviews were conducted between June and August 2021 with five resident physicians and all five of the institution’s ICU specialty pharmacists. Interview length ranged from 28 min to 58 min with an average length of 48 min. The physician participants in the study came from the following years in their residency program: year 1 (N = 1), year 2 (N = 3), and year 3 (N = 1). The pharmacy participants worked across multiple ICUs but each specialized in one or more of the following ICUs: medical ICU, trauma-surgical ICU, cardiac ICU, and neuro ICU. All participants performed daily work in ICUs throughout the duration of the study and had regular interactions with patients and other practitioners regarding antimicrobial therapies.

### 3.1. Limited Autonomy

Residents and pharmacists involved in the study characterized their roles within the hospital hierarchy as providing assistance to attending physicians for individual patient cases. As summarized in Table 1, a common theme that emerged was a sense of limited autonomy. Residents noted that their role involved doing “whatever the attending says”. The principal decision-makers related to antimicrobials were seen to be the attending physicians, who might “dictate” or “make the final call” for patient care. Similarly, pharmacists acknowledged their role as one of consultation on patient cases. Pharmacist 2 stated, “[The physicians] are ultimately responsible.” Neither residents nor pharmacists were under the impression that they had a meaningful level of autonomy over patient cases or antimicrobial decision-making.

At the same time, the pharmacist participants described methods of navigating the hospital hierarchy such that when they noticed that a physician wanted to “cover everything” without regard for antimicrobial stewardship, it was possible to steer the decision-making from a consultant’s position. This occasionally necessitated support from antimicrobial stewardship or infectious diseases team members. For example, one pharmacist participant stated, “Sometimes you need to phone a friend that has a little more power than you have”. Thus, while participants sensed they were not the primary decision-makers related to antimicrobial use or infectious diseases management, at times they were able to advocate for the principles of antimicrobial stewardship given a strong network of colleagues and established channels of communication.

### 3.2. Overtreatment and Instant Gratification

Another theme which emerged was the concept of precautionary treatment and the associated sense of reward (Table 2). Despite experiencing limited autonomy in the clinical setting, both resident physicians and pharmacists described the impetus to intervene in patient cases where infection was likely or possible. Participants explained that even when antimicrobial use is added to a regimen early or multiple antimicrobials are prescribed prior to evidence of infection, the “safety blanket” of antimicrobials provide a strong draw. After all, participants explained, antimicrobials can “save a lot of lives” and ultimately “eradicate the infection”. The theme of overtreating to be safe saturated the data set such that every participant referred to the importance of adding an antimicrobial to a patient’s treatment plan. At the same time, some participants recognized the potential for excessive antimicrobial use. This was exemplified by one participant who stated that sometimes the use of antimicrobials was like “shooting an ant with an elephant gun”.

The threat of infection, particularly antimicrobial-resistant infection, was so high in the minds of residents and pharmacists that the prescription of antimicrobials gave participants a sense of “immediate gratification”. Providing intensive care was described as “rewarding,” and the urgency of initiating antimicrobials was repeatedly mentioned by participants. Even when participants noted the mismatch between the mere potential of infection and the massive arsenal of antimicrobials utilized, the compulsion to provide instant treatment overpowered the incentive to advocate and intervene from a stewardship perspective. In the context of critical care and anesthesia as professional fields, the participants suggested that there is a strong draw towards actions that would take immediate effect. With regards to how this draw impacts antimicrobial use, the practice of prescribing antimicrobials appeared to be impacted by the overall mindset of the participants who were working in intensive care units.

## 4. Discussion

Resident and pharmacist participants in this study were largely in agreement that their role allowed for limited contributions to antimicrobial discussions, decision-making, and prescription, thereby restricting their ability to have autonomy over antimicrobial use. Participants tended to speak about their role in relationship to a broader hospital hierarchy of practice that placed attending physicians and administrators at a higher level than physicians in training and non-physician practitioner staff. This finding sits in relation to a second major theme: overtreating to be safe and instant gratification. Residents and pharmacist participants expressed a need to treat early for some patients and to cover the potential of infection given the threat of antimicrobial-resistant infections. Participants in this study had an empathic approach to understanding antimicrobial use. Thus, while participants regretted their limited autonomy over antimicrobial use, they also were understanding of the rationales and value of antimicrobial use in hospital settings.

Overtreating to be safe relates to several tropes in studies of antimicrobial use. Primarily, if microbes such as bacteria are seen as threatening, a strong impetus to protect exists in medical care around infection. As a safety issue, the use of antimicrobials as a “magic bullet” or “just in case”-prescribing can thus easily lead to overtreatment [15,16,17]. The concept of the magic bullet was famously used by biochemist Paul Ehrlich to describe Salvarsan 606′s effect on syphilis and became a commonly utilized frame for medicinal use in human populations [18,19]. Here, antimicrobials can be seen as magic bullets as they provide protection but also serve to eradicate organisms, an action that ultimately can create a domino effect within a patient’s microbiome. While an awareness of the dangers of antimicrobial use is growing, it appears that the habits of overtreatment are closely tied to patterns of medical practice unrelated to antimicrobials [20,21,22].

Instant gratification as a theme draws on literature that frames antimicrobials as technological solutions to medical problems [23]. Namely, antimicrobials as quick-fix solutions [24] reflect pervasive ideas about what antimicrobials are and what they can be used for [25,26]. While overtreating to be safe depicts the pressures of safeguarding patients and avoiding medical mistakes, instant gratification as a theme can also refer to the feeling a physician gets when they solve a patient case. For example, a physician may feel that the sooner a patient is out of the woods, the sooner they as caregiver can relax and settle into a treatment plan. In a field with high rates of burnout [27,28], understanding what pressures and feelings physicians experience can help explain why leaving the field could be a viable option. Antimicrobial prescribing as a practice thus appears to have potential impacts not only on the patient but also on the prescriber.

The implications of this work are numerous. Building upon discussion of empowering practitioners in their positions within the hospital-based hierarchy [29,30,31], we conclude that just as AMR has an impact spanning hierarchy and institutional bounds, antimicrobial stewardship should acknowledge the multifaceted nature of medical practice in intensive care unit patients. Antimicrobial stewardship was described in a broad and programmatic sense by participants. The threat of antimicrobial resistance was also perceived by participants, albeit distantly. Even given the hesitancy to intervene in cases of antimicrobial overuse and misuse, participants were open to the view that antimicrobial use could be better tailored to patient cases. Thus, the study found that antimicrobial stewardship is a topic the participants are willing to discuss and learn more about.

Antimicrobial stewardship is a critical intervention for the global public health issue of AMR, and well-established evidence has shown that antimicrobial stewardship has the capacity to reduce antimicrobial use in medical settings, including ICUs [3,32,33]. However, the exact circumstances and types of practitioners required for this impact to be visible in practice norms are unclear. Both residents and pharmacists described a high level of involvement with daily antimicrobial prescribing practice, in addition to explaining their beleaguered position in relation to having autonomy over antimicrobial decisions. The qualitative results of this study suggest that clinical teams such as those involved in our study are integral to the utilization of antimicrobials in intensive care units. Practitioners who are involved in discussing, documenting, calculating, and administering antimicrobials should also be considered decision-makers and could additionally be folded into existing antimicrobial stewardship programming. Residents and pharmacists in this study did have a broad appreciation for the principles of antimicrobial stewardship, which may mean that this population is a valuable yet underutilized resource for improving antimicrobial prescribing on the front lines.

In this study, we did not aim to develop or test interventions. This represented a qualitative investigation that focused on in-depth interview data, and a major strength of these data is the captured rationales and experiences of practitioners. Our limitations are related to the specificity of residency training and pharmacy specialty of the institution as well as absence of in-person observation which would have enriched the interview data. These results are not representative of the diverse range of institutions present in the United States; neither do we claim that all pharmacists or all residents would have responded similarly. Instead, the selected sample included is representative of ICU pharmacists and residents who are not infectious diseases specialists at this academic medical center.

In the consideration of future studies, it is notable that only ICU practitioners who were rotating or working during the study period were interviewed. Furthermore, multiple practitioners worked across more than one ICU, and thus, interview data could not be used to draw comparison between ICUs. Finally, while the interview data were in-depth and several themes emerged from the data, a follow-up period of observation to confirm themes in practice was not possible given institutional constraints and reasonable infection control precautions. Future studies could consider an ethnographic approach that facilitates both interviews and observation to provide a two-pronged understanding of rationales and practice. Additionally, because antimicrobials are used across all ICU settings a comparative study of practitioners at all levels and in all ICU settings could be a valuable approach to bolstering antimicrobial stewardship programming.

## Figures and Tables

**Table 1:** Exemplar quotes pertaining to the theme of limited autonomy.

Exemplar Quotes
If we’re changing it [antibiotics], then...the orders would be putting it in at that time based on what the discussion is with the attending...realistically, just whatever the attending says. (Resident 1)
Usually I’m the one providing that [antibiotic information] to the surgeon, saying ‘the person was already on...whatever they were on...do you want to continue with these same antibiotics’...kind of surgeon preference. (Resident 1)
Most of the time they [the ICU teams] are willing to consult ID, but sometimes...they just want to order something, and we’ll have to be the intermediary. (Pharmacist 2)
The [ICU] teams are pretty receptive to me when I recommend...but sometimes you get people that are more receptive than others. (Pharmacist 4)
The surgical ICU is open, so surgeons come in and do stuff with their residents and do not always tell us. It’s frustrating because they’re ultimately responsible for the patient care while they’re in the unit...it can be a point of contention. (Pharmacist 2)
I’d probably ask an attending who knows more about antibiotics than I do...if nobody has any answers [to antibiotic questions] or if it’s a tricky situation, we consult ID. (Resident 5)
We’re in a unique position because we round with the team and we’re there for the discussion…and I know how to reach the stewardship pharmacist and escalate the situation so that it can be taken care of by ID. (Pharmacist 2)
I’m not usually the one making that final call. And if I was, I probably would be doing the same thing because I think there’s a fear of undertreating the patient.” (Resident 1)
It can be difficult if a team really wants to continue therapy and the ID [infectious diseases] team or the pharmacist wants to deescalate. “No, I want to keep them on this because I’m more comfortable with it. I want to just cover everything ”...Well, sometimes you need to phone a friend that has a little more power than you have, and that’s the ID attending that’s responsible for the stewardship team. (Pharmacist 2)
It seems based on hearsay, sometimes practice changes...Some physicians have their own preference... so there’s some non-evidence-based thoughts in practice. Whether the physician is trying to do that because they had a patient that failed [treatment] or sometimes, they try to avoid drugs that require levels because they’re more labor-intensive. (Pharmacist 3)
Some attendings that I worked with were way more comfortable with their antibiotic management and others were like just call ID. So, again, it’s not really up to us so much as the higher powers. (Resident 1)
I should probably give more thought about what I’m using. Unfortunately, I don’t have any more time so the reflection would come later after rounding. If the attending, you know, admonished me for not thinking it through. (Resident 1)

Abbreviations: ICU, intensive care unit; ID, infectious diseases.

**Table 2:** Exemplar quotes pertaining to themes of overtreatment and instant gratification.

Exemplar Quotes
**Overtreatment**
People lean very heavily towards treating the patient first because there’s a fear of undertreating the patient. (Resident 1)
If you’re on a border between adjusting an [antibiotic] dose or leaning higher, you want to go a little bit on the high side because we have to make sure we eradicate the infection rather than worry about possible accumulation of an antibiotic that has lower risk for causing toxicity anyway. (Pharmacist 2)
We usually start fairly strong and then de-escalate...kind of the pattern. (Resident 4)
When there’s an active infection that we don’t know about or when the surgeons are not sure exactly what they’re going to be doing...they’ll want something a little broader. (Resident 1)
We’re trying to cover for as much as possible, as quickly as possible. Once we find out what they [the bacteria] are sensitive to, then we can narrow it. I’d say upon first presentation of infectivity or them being sick, it [stewardship] is not as important. Eventually we will try to be antimicrobial stewards once we get a better idea of what we’re dealing with, infection wise. (Resident 3)
But giving antibiotics is one of the easiest things that we can do, and it saves a lot of lives. We focus on just empirically treating but then pulling back when you know that it’s not an infection. (Pharmacist 3)
We err on the side of caution and give them medications that will cover more antibiotics [organisms]...so we can prevent their infection from getting worse. (Resident 3)
As far as other broad-spectrum antimicrobials it’s kind of like shooting an ant with an elephant gun. It seems sometimes like it’s potentially too broad...but it’s a rather better to be safe than sorry versus thinking whether it’s appropriate or not. (Pharmacist 5)
With antibiotics in general we tend to use broader agents. It doesn’t take a lot to trigger us to use it which is fair because you get very sick people and if you ignore a potential infection, it can definitely get worse. (Pharmacist 4)
We don’t have a great explanation [for unnecessary prescriptions]...it’s like checking off a box...it’s like a diagnostic tool in most cases, which is probably inappropriate. (Resident 1)
The pattern has always been if you have a patient with sepsis and an unknown cause and you put them on broad-spectrum [antibiotics]. Then get blood cultures and narrow after sensitivities have come back and then figure out the stop dates after that. (Resident 5)
The safest way would be to put them on something broad to cover most things and then figure it out afterwards when you have cultures back. It’s an appropriate way to use antibiotics. (Resident 5)
You can’t assume what bug a person is growing...So, starting broad, trying to get as many bugs as possible, and then narrowing down to a specific bug. I think that’s kind of the way I think you should do it. (Resident 2)
In the ICU they could be infected with anything. So unfortunately, it’s our safety blanket to use these antibiotics as soon as our patients give any signs that they are infected, because if we leave their infections untreated, then they could devolve very, very quickly. (Resident 3)
**Instant Gratification**
The ICU patients are so ill that it gives you great satisfaction if you can help the team with the therapeutic decision that impacts someone’s chances of getting better quickly…that’s very rewarding. (Pharmacist 2)
I don’t know if there’s always such a clear-cut path to being good antibiotic stewards because of that like imperative to treat your patients and make sure that this individual patient, you know, recovers from their infection. It’s easier to think about them than to think about the hypothetical patient in the future with a resistant organism. (Resident 1)
I found it more satisfying to, like, help a surgical patient along from the perspective of anesthesia so you could see them have immediate benefit to your interventions…we get to deal with a lot of medications and clinical interventions…I like all the interventions. (Resident 3)
Sometimes their cultures wouldn’t result anything. But then we would keep them on antibiotics for several days because we saw that they were clinically improving...instead of a seven-day course, let’s just make it a three-day course. (Resident 3)
With anesthesia one thing is...it’s very quick and immediate. And I know in our generation it’s like immediate gratification...I kind of like how there’s a problem and you can quickly fix it and I like that aspect of it. (Resident 2)

Abbreviations: ICU, intensive care unit.

## Data Availability

The data presented in this study are available on request from the corresponding author. The data are not publicly available due to privacy and confidentiality concerns.

## References

[1] World Health Organization (2015). Global Action Plan on Antimicrobial Resistance.

[2] Centers for Disease Control and Prevention (2019). Antibiotic Resistance Threats Report.

[3] Nathwani D., Varghese D., Stephens J., Ansari W., Martin S., Charbonneau C. (2019). Value of hospital antimicrobial stewardship programs [ASPs]: A systematic review. Antimicrob. Resist. Infect. Control.

[4] Dyar O., Huttner B., Schouten J., Pulcini C. (2017). What is antimicrobial stewardship?. Clin. Microbiol. Infect..

[5] Meeker D., Knight T., Friedberg M., Linder J., Goldstein N., Fox C., Rothfeld A., Diaz G., Doctor J.N. (2014). Nudging guideline-concordant antibiotic prescribing. JAMA Intern. Med..

[6] Charani E., Castro-Sánchez E., Holmes A. (2014). The Role of Behavior Change in Antimicrobial Stewardship. Infect. Dis. Clin. N. Am..

[7] Barlam T., Childs E., Zieminski S., Meshesha T., Jones K., Butler J., Damschroder L., Bidwell Goetz M., Madaras-Kelly K., Reardon C. (2020). Perspectives of Physician and Pharmacist Stewards on Successful Antibiotic Stewardship Program Implementation: A Qualitative Study. Open Forum Infect. Dis..

[8] Carter E., Greendyke W., Furuya E., Srinivasan A., Shelley A., Bothra A., Saiman L., Larson E. (2018). Exploring the nurses’ role in antibiotic stewardship: A multisite qualitative study of nurses and infection preventionists. Am. J. Infect. Control.

[9] Kollef M.H., Bassetti M., Francois B., Burnham J., Dimopoulous G., Garnacho-Montero J., Lipman J., Luyt C.E., Nicolau D.P., Postma M.J. (2017). The intensive care medicine research agenda on multidrug-resistant bacteria, antibiotics, and stewardship. Intensive Care Med..

[10] Bernard H. (2018). Research Methods in Anthropology: Qualitative and Quantitative Approaches.

[11] Lincoln Y., Guba E. (1985). Naturalistic Inquiry.

[12] Sandelowski M. (1995). Sample size in qualitative research. Res. Nurs. Health.

[13] Pope C. (2005). Conducting ethnography in medical settings. Med. Educ..

[14] Saldaña J. (2021). The Coding Manual for Qualitative Researchers.

[15] Sengupta S., Chattopadhyay M.K., Grossart H.P. (2013). The multifaceted roles of antibiotics and antibiotic resistance in nature. Front. Microbiol..

[16] Lucas P.J., Cabral C., Hay A.D., Horwood J. (2015). A systematic review of parent and clinician views and perceptions that influence prescribing decisions in relation to acute childhood infections in primary care. Scand. J. Prim. Health Care.

[17] Pandolfo A.M., Horne R., Jani Y., Reader T.W., Bidad N., Brealey D., Enne V.I., Livermore D.M., Gant V., Brett S.J. (2021). Understanding decisions about antibiotic prescribing in ICU: An application of the Necessity Concerns Framework. BMJ Qual. Saf..

[18] Ehrlich P., Himmelweit F. (1960). Address delivered at the Dedication of the Georg-Speyer-Haus. The Collected Papers of Paul Ehrlich.

[19] Gradmann C. (2011). Magic bullets and moving targets: Antibiotic resistance and experimental chemotherapy, 1900–1940. Dynamis.

[20] Kamzan A.D., Ng E. (2021). When Less is More: The Role of Overdiagnosis and Overtreatment in Patient Safety. Adv. Pediatr..

[21] Armstrong N. (2021). Overdiagnosis and overtreatment: A sociological perspective on tackling a contemporary healthcare issue. Sociol. Health Illn..

[22] Lyu H., Xu T., Brotman D., Mayer-Blackwell B., Cooper M., Daniel M., Wick E.C., Saini V., Brownlee S., Makary M.A. (2017). Overtreatment in the United States. PLoS ONE.

[23] Tarrant C., Krockow E.M., Nakkawita W.M.I.D., Bolscher M., Colman A.M., Chattoe-Brown E., Perera N., Mehtar S., Jenkins D.R. (2020). Moral and Contextual Dimensions of “Inappropriate” Antibiotic Prescribing in Secondary Care: A Three-Country Interview Study. Front. Sociol..

[24] Denyer Willis L., Chandler C. (2019). Quick fix for care, productivity, hygiene, and inequality: Reframing the entrenched problem of antibiotic overuse. BMJ Glob. Health.

[25] Khare S., Pathak A., Stålsby Lundborg C., Diwan V., Atkins S. (2022). Understanding Internal and External Drivers Influencing the Prescribing Behaviour of Informal Healthcare Providers with Emphasis on Antibiotics in Rural India: A Qualitative Study. Antibiotics.

[26] Reynolds Whyte S., van der Geest S., Hardon A. (2009). Social Lives of Medicines.

[27] Pastores S.M., Kvetan V., Coopersmith C.M., Farmer J.C., Sessler C., Christman J.W., D’Agostino R., Diaz-Gomez J., Gregg S.R., Khan R.A. (2019). Workforce, Workload, and Burnout Among Intensivists and Advanced Practice Providers: A Narrative Review. Crit. Care Med..

[28] Gualano M.R., Sinigaglia T., Moro G.L., Rousset S., Cremona A., Bert F., Siliquini R. (2021). The Burden of Burnout among Healthcare Professionals of Intensive Care Units and Emergency Departments during the COVID-19 Pandemic: A Systematic Review. Int. J. Environ. Res. Public Health.

[29] Schmid S., Schlosser S., Gülow K., Pavel V., Müller M., Kratzer A. (2022). Interprofessional Collaboration between ICU Physicians, Staff Nurses, and Hospital Pharmacists Optimizes Antimicrobial Treatment and Improves Quality of Care and Economic Outcome. Antibiotics.

[30] Wong L.H., Tay E., Heng S.T., Guo H., Kwa A.L.H., Ng T.M., Chung S.J., Somani J., Lye D.C.B., Chow A. (2021). Hospital Pharmacists and Antimicrobial Stewardship: A Qualitative Analysis. Antibiotics.

[31] Preslaski C.R., Lat I., MacLaren R., Poston J. (2013). Pharmacist Contributions as Members of the Multidisciplinary ICU Team. Recent Adv. Chest Med..

[32] Huebner C., Flessa S., Huebner N.O. (2019). The economic impact of antimicrobial stewardship programmes in hospitals: A systematic literature review. J. Hosp. Infect..

[33] Karanika S., Paudel S., Grigoras C., Kalbasi A., Mylonakis E. (2016). Systematic Review and Meta-analysis of Clinical and Economic Outcomes from the Implementation of Hospital-Based Antimicrobial Stewardship Programs. J. Antimicrob. Agents Chemother..

